# The Critical Role of Tryptophan in the Antimicrobial Activity and Cell Toxicity of the Duck Antimicrobial Peptide DCATH

**DOI:** 10.3389/fmicb.2020.01146

**Published:** 2020-05-28

**Authors:** Xingjun Feng, Sanjun Jin, Min Wang, Qian Pang, Chunlong Liu, Ruiqi Liu, Yingjie Wang, Hao Yang, Fangju Liu, Yueying Liu

**Affiliations:** ^1^Institute of Animal Nutrition, Northeast Agricultural University, Harbin, China; ^2^Northeast Institute of Geography and Agricultural Ecology, Chinese Academy of Sciences, Harbin, China; ^3^College of Pharmaceutical Science, Zhejiang University of Technology, Hangzhou, China

**Keywords:** antimicrobial peptides, cathelicidin, tryptophan, cell selectivity, antimicrobial mechanism, lipopolysaccharide, mice

## Abstract

Antimicrobial peptides (AMPs) have attracted more attention for their potential candidates for new antibiotic drugs. As a novel identified cathelicidin AMP from duck, dCATH owns broad-spectrum antimicrobial activities but with a noticeable toxicity. To explore dCATH-derived AMPs with reduced cell toxicity and improved cell selectivity, a series of truncated and tryptophan-replaced peptides of dCATH were designed. Two truncated peptides containing one of the two tryptophan (Trp) residues at the positions of 4 and 17 (W4 and W17) of dCATH, dCATH(1–16) and dCATH(5–20), showed strong antibacterial activity, but didn’t show obvious hemolytic activity and cytotoxicity. The derived peptides not containing Trp didn’t possess obvious antimicrobial activity, and their hemolytic and cytotoxic effect was also diminished. Also as evidence by Trp fluorescence experiment that existence of W4 and W17 was crucially important to the antimicrobial activity, hemolysis and cytotoxicity of dCATH, and one of the two Trp residues was competent and necessary to retain its antimicrobial activity. Antibacterial mechanism analysis showed that dCATH(1–16) and dCATH(5–20) killed bacterial cells by increasing permeability and causing a loss of membrane integrity. dCATH(1–16) and dCATH(5–20) possessed insignificant inhibitory activity against levels of IL-6, TNF-α, and NO in RAW 264.7 cells treated with LPS. *In vivo*, intraperitoneal administration of the two peptides significantly decreased mortality and provided protection against LPS-induced inflammation in mice challenged with lethal dose of LPS. The two peptides, dCATH(1–16) and dCATH(5–20), which possessed high antibacterial activity and cell selectivity, may herald development prospects as new antibacterial agents in the future.

## Introduction

In recent years, the frequent emergence of drug-resistant microorganisms has become a threat to public health and safety (Wang et al., 2019). The number of multidrug-resistant bacteria is in a rapid increase (Zumla et al., 2014). There is a pressing need to develop new antimicrobial agents that are active against bacteria and unlikely to cause resistance. Among the possible candidates, AMPs have elicited increasing interest, because AMPs have broad spectrum of antimicrobial activity and Low probability of bacterial resistance (Teixeira et al., 2012). Exact mechanism of AMPs action has not been fully elucidated, but most cationic AMPs possess non-specific activities of membrane lysis, which are more difficult for bacteria to produce resistance than to conventional antibiotics (Dong et al., 2018; Ho et al., 2019).

Currently, more than 2,000 AMPs have been characterized from natural sources (Wang et al., 2009). As one of two families of animal AMPs (McGillivary et al., 2007), cathelicidins (CATHs) have been identified in almost all kinds of vertebrates (Nakamichi et al., 2014). CATHs play an important role in the innate immune of organisms (Zanetti, 2004). Full length CATHs are in form of holoproteins, including a signal peptide, highly conserved cathelin domain and functional mature peptide at C-terminus with variable sequences (Zanetti et al., 1997). The mature CATHs at the C-terminus express their bioactivity, such as antimicrobial activity and immune regulatory activities, after they were cleaved from the holoprotein. Current evidences indicate that they exhibit antimicrobial activity against many kinds of microorganisms including fungi, bacteria and enveloped viruses, the ability to neutralize endotoxin, immunomodulating property, etc. (Maroti et al., 2011; Khurshid et al., 2017; Zhang et al., 2019).

Cathelicidins have been regarded as potential alternatives of novel antimicrobial agents in recent years. However, their development used as antimicrobial agents is presently limited by some dilemmas. The antimicrobial activity of most AMPs can be suppressed by salt or serum (Lee et al., 1997; Ciornei et al., 2005). In addition, the noticeable toxicity, such as hemolysis and cytotoxicty to the host cells is associated with many AMPs. Various means have been developed for designing novel AMPs with strong antimicrobial activity, good stability and week cytotoxicity, such as motif hybridization (Feng et al., 2014), *de novo* (Deslouches et al., 2005; Rydberg et al., 2012), and substitution/truncation (Jacob et al., 2013; Cao et al., 2015; Qu et al., 2016). The partial sequence of natural AMPs can retain the antimicrobial activity and decrease the hemolytic activity. Truncation of the natural AMPs is an effective way for exploring a novel candidate of AMPs (Jacob et al., 2013; Jittikoon et al., 2015; Qu et al., 2016). Furthermore, the synthesis cost of short peptides will also be decreased due to the number reduction of amino acid residues.

We have found a new CATH AMP from duck, dCATH, which is composed of 20 amino-acid acids (KRFWQLVPLAIKIYRAWKRR), and owns broad-spectrum antimicrobial activity, meanwhile has noticeable cytotoxicity to the host cells (Gao et al., 2015). To reduce the cell toxicity and develop novel AMPs with high cell selectivity, we got a series of dCATH derivatives by truncating from the N-terminal or C-terminal of the peptide. The antibacterial activities of these peptides were investigated by measuring the minimal inhibitory concentrations (MICs) against the selected threatening bacteria. Their activities of hemolysis and cytotoxicity were also determined. Potential membrane destruction mechanisms were investigated employing bacteria. Changes of cell morphology treated with peptide were observed by scanning electron microscopy (SEM) and transmission electron microscopy (TEM). Finally, effect of peptides on LPS-induced inflammation was investigated *in vitro* and *in vivo*.

## Materials and Methods

### Design and Synthesis of the Peptides

At the amino and carboxyl terminus of the peptide dCATH were aggregated several amino acids, such as W, Lys (K), Arg (R), which are usually important to the activity of AMPs. To investigate the influence of these amino acids on the activity of the peptide dCATH, six peptides were designed by truncation from the N or C-terminus of dCATH and chemically synthesized. To determine the effect of W4 and W17 on the antibacterial, hemolytic and cytotoxic activity of dCATH, three peptide analogs, dCATH-AA, dCATH(1–16)-4A, and dCATH(5–20)-17A, in which W4 and W17 were replaced by the Ala residue, were also designed and synthesized. A shorter peptide only containing the 12 amino acid residues in the middle of dCATH, dCATH(5–16), was also synthesized to investigate the role of the fragment sequence. These peptides were synthesized through chemical method (Shanghai Ltd., Shanghai, China). The true molecular weights were determined by electrospray ionization mass spectrometry (ESI-MS), and the purities (>95%) were assessed by reversed phase high performance liquid chromatography (RP-HPLC). The primary physicochemical parameters were determined using bioinformatics software including ProtParam (ExPASyroteomics Server)^1^ and the AMPs database^2^. The three-dimensional structure predictions were carried out online using I-TASSER^3^.

### Bacterial Strains and Animal Cells

Three types of Gram-positive bacteria (*Staphylococcus epidermidis* ATCC 12228, *Staphylococcus aureus* 29213 and *Enterococcus faecalis* ATCC 29212) and four types of Gram-negative bacteria (*Escherichia coli* UB1005, *Escherichia coli* ATCC 25922, *Salmonella typhimurium* ATCC 14028 and *Salmonella pullorum* C79–13) preserved in our lab were cultured in the medium of Muller-Hinton broth (MHB) medium (1.75% casamino acid, 0.2% beef extract, 0.15% soluble starch, pH 7.0). The murine macrophage cell line RAW 264.7 cells were procured from the American Type Culture Collection (Manassas, VA, United States) and cultured in DMEM containing 10% fetal bovine serum (BSA), penicillin (100 units/ml) and streptomycin (100 units/ml) in 5% CO_2_ at 37°C.

### Hemolytic Activity Assay

The hemolytic activity assay of these peptides was performed according to the method of Gao et al. (2015). Human red blood cells (hRBCs) donated by a healthy adult were collected by centrifugation and washed by phosphate-buffered saline (PBS) solution (pH 7.2). 1% (v/v) suspension of hRBCs in PBS was incubated with different concentrations of peptides dissolved in PBS for 1 h at 37°C. Absorbance in the supernatant was monitored at 405 nm. hRBCs in 0.1% Triton X-100 and PBS were used as positive and negative controls, respectively. The hemolysis percentage was calculated as follows:

%hemolysis=[A-sampleA]blank/[A-TritonA]blank×100.

### Cytotoxicity Assay

The colorimetric 3-[4,5-dimethylthiozol-2-yl]-2,5-diphenyltetrazolium bromide (MTT) can be reduced by succinate dehydrogenase in the mitochondria of living cells to the water-insoluble formazan crystals with blue purple and deposited in cells, but the dead cells do not have this function. The peptide cytotoxicity was determined by the colorimetric MTT dye reduction assay as a previously described method (Xiao et al., 2009). The RAW 264.7 cell suspension was mixed with aliquots of peptide solutions in 96 well plates (the final concentration range of peptide was 1–128 μm). After being incubated in a totally humidifying environment of 5% CO_2_ at 37°C for 18–24 h, the cells were further cultured with 50 μl of MTT (0.5 mg/ml) at 37°C for 4 h. To dissolve the formazan crystals formed, dimethyl sulfoxide (DMSO) (150 μl) was added. Finally, absorbance at 570 nm was determined by a microplate reader (TECAN, Switzerland). The cells not treated with peptide were used as control. The following equation was used to calculate cell viability:

(A⁢of570⁢treated⁢sample)/(A⁢of570⁢control)×100%.

### Antimicrobial Activity Assay

The antimicrobial activity of peptides was examined against representative strains of Gram-positive bacteria (*S. aureus* ATCC 29213, *S. epidermidis* ATCC 12228 and *E. faecalis* ATCC 29212) and Gram-negative bacteria (*E. coli* ATCC 25922, *E. coli* UB1005, *S. pullorum* C79–13, and *S. typhimurium* ATCC 14028) which were cultured in MHB medium. Antimicrobial activity assay was performed according to a modified version of broth microdilution method (Gao et al., 2015). When grown to an OD_600_ of 0.4, the bacterial stains cultured in MHB were diluted to a final concentration of about 1 × 10^5^ CFU/ml. Fifty microliter of microbial solution and 50 μl of two-fold diluted concentrations of peptides [0.2% (w/v) bovine serum albumin, 0.01% (v/v) acetic acid] were mixed in the sterile 96-well plate and cultured at 37°C for 24 h. MICs were identified as the lowest peptide concentration that no bacteria growth was observed by OD determination (492 nm). Each experiment was performed in triplicate. The geometric mean (GM) of the MICs of a peptide was calculated on the MIC values of the peptide against all tested bacteria, and was used to evaluate the antimicrobial activity of the peptide. The therapeutic index (TI) was defined as the ratio of the minimum hemolytic concentration (MHC) to the GM and was used to measure the peptide cell selectivity toward the component with negative charge of bacterial cell membranes over the zwitterionic membranes mammalian cells. TI was calculated as the ratio of the HC10 (the peptide concentration needed to reach 10% lysis of hRBCs) value to the GM (TI = HC10/GM).

### Salt Sensitivity Assay

For salt sensitivity assay, the MIC values of peptides were tested using a modified method (Maisetta et al., 2008; Chou et al., 2016). *S. aureus* ATCC 29213 and *E. coli* ATCC 25922 were cultured in the medium containing different salts at their physiological concentrations (150 mM NaCl, 4.5 mM KCl, 2.5 mM CaCl_2_, 1 mM MgCl_2_, 8 μM ZnCl_2_, 6 μM NH_4_Cl, and 4 μM FeCl_3_). MIC values were determined after 24 h, and MIC values without physiological salt addition were as control.

### Preparation of Small Unilamellar Vesicles (SUVs)

Small unilamellar vesicles were prepared with either phosphatidylcholine (PC)/phosphatidylglycerol (PG) (7:3, w/w) or PC/cholesterol (CH) (8:1, w/w) as described previously (Zhang et al., 2001). An appropriate amount of dried lipid was dissolved in chloroform, dried under nitrogen flow, and frozen dry overnight. Dried lipid films were resuspended in PBS (10 mM, pH 7.4), and then sonicated in ice water for 40 min until the solution became transparent.

### Trp Fluorescence Blue Shift

Trp fluorescence experiment was carried out to assess the peptide-lipid interaction (Lee et al., 2014). The fluorescence spectroscopy analysis of the Trp residues in peptide was conducted in HEPES buffer (150 mM NaCl, 10 mM HEPES, pH 7.4) as well as in the presence of PE/PG SUVs or PC/CH SUVs at 10 μM of peptide (lipid mixture with molar ratio of 1:100). After being incubated at 25°C for 10 min, the fluorescence spectrum of tryptophan was determined by an F-4600 fluorescence spectrophotometer (Hitachi, Japan). The excitation of wavelength was set as 280 nm, and the emission spectra of each peptide were recorded between 300 and 400 nm. The blue shift of Trp residue in vesicles was calculated by subtracting the fluorescence spectrum of the peptide-liposomes from the liposomes spectrum alone.

### Cytoplasmic Membrane Depolarization Assay

A membrane potential-sensitive fluorescent dye, 3,3-dipropylthiadicarbocyanineiodide (diSC_3_-5), was used to determine the degree change of cytoplasmic membrane potential (Gao et al., 2015). *S. aureus* ATCC 29213 and *E. coli* ATCC 25922 were served to measure the ability of cytoplasmic membrane depolarization of dCATH(1–16) and dCATH(5–20). When growing to the mid-log phase, bacterial cells were washed and resuspended in buffer (5 mM HEPES, 5 mM glucose, pH 7.4) to an OD_600_ of 0.05. 0.4 mM diSC_3_-5 was added into the cell suspension (OD_600_ = 0.05) and incubated for 90 min. 0.1 M concentration of K^+^ was added, and incubated at room temperature for 15–30 min. Membrane depolarization was determined after peptide addition over a period of 600s. Fluorescence was measured by fluorescence spectrophotometer (TECAN, Austria), with the excitation and emission wavelength being set at 670 and 622 nm, respectively.

### Membrane Permeability Assay

*Staphylococcus aureus* ATCC 29213 and *E. coli* ATCC 25922 were used to determine membrane permeabilization ability of peptides based on the cytoplasmic β-galactosidase activity in bacterial cells using ONPG (**o**-Nitrophenyl-β-D-Galactopyranoside) as the substrate, which has been previously described (Dong et al., 2018). ONPG is blocked from entering bacterial cells by the cell membrane. When the bacterial membrane permeability occurred, ONPG is able to traverse this barrier and be hydrolyzed by β-galactosidase within the cytoplasm, which results in yellow color appearance. After being harvested and washed twice, bacteria in mid-log phase was diluted in 10 mM PBS buffer containing 1.5 mM ONPG (pH 7.4) to an OD_600_ of 0.05. The bacteria were treated with different peptides at the concentrations of 1 × MIC and 1/2 × MIC. The hydrolysis of ONPG to *o*-nitrophenol over time was monitored and determined by spectrophotometer (TECAN GENios F129004; TECAN, Austria) at 420 nm from 0 to 60 min every 5 min after peptide addition. All assays were repeated in triplicate.

### SEM

As previously described (Ramanathan et al., 2002), *E. coli* ATCC 25922 was harvested and resuspended with PBS (10 mM) when growing to exponential phase. The bacterial cells (OD_600_ = 0.2) were treated with 1 × MIC peptides at 37°C for 1 h, centrifuged and washed 3 times with PBS. Bacterial cells were harvested by centrifugation, and fixed in 500 ml of PBS containing 2.5% (v/v) glutaraldehyde at 4°C for one night. After being washed twice with PBS, the bacterial cells were dehydrated by a graded series of ethanol (50, 70, 90, and 100%) for 15 min in each dilution. The cells were treated with a mixture of ethanol and tertiary butanol (1:1, v/v) and pure tertiary butanol for 20 min for each step, respectively. After freeze-drying and gold coating, the bacterial cells were observed under a scanning electron microscope (Hitachi S-4800, Hitachi, Japan).

### TEM

Bacterial sample pre-treatment was the same as that of SEM. After glutaraldehyde fixation and being washed twice with PBS, the bacterial sample was fixed with 2% osmium tetroxide for 80 min. Bacterial cells were dehydrated with a graded series ethanol (50, 70, 90, and 100%) for 8 min in each step, and then incubated in 100% ethanol, mixture of ethanol and acetone (1:1), and 100% acetone for 10 min each. The cells were then immersed in a 1:1 mixture of acetone and epoxy resin for 30 min, and in a pure epoxy resin overnight. After being sliced into ultraslices and stained in uranylacetate and lead citrate, the cells were observed under a HITACHI H-7650 TEM.

### LPS Binding Assay

LPS binding assay was carried out to detect peptide-LPS interaction using BODIPY-TR-cadaverine fluorescent dye (BC, Sigma, United States) as probe (Sautrey et al., 2014), where BC fluorescence can be quenched by the binding of LPS and peptide. A mixture of LPS (*E. coli* 055: B5, 100 ng/ml) and BC (5 μg/ml) in Tris buffer (50 mM, pH 7.4) was incubated at room temperature for 4 h. Serially concentration dilutions of peptide (1–64 μg/ml) was mixed with an equal volume of the LPS-BC mixture and incubated at 37°C for 1 h. The fluorescence was measured using a spectrofluorophotometer (the Infinite 200 pro, Tecan, China) with excitation λ = 580 nm and emission λ = 620 nm. The values were converted to %ΔF (AU) as follows:

%ΔF(AU)=[(F-obsF)0/(F-100F)0]×100

where F*_*obs*_*, F_0_, and F_100_ were the fluorescence values of a given peptide concentration, LPS with BC, and LPS by adding polymyxin B (PMB) (10 μg/ml), respectively. The assay was conducted three times with replicates.

### Inhibition of Pro-inflammatory Mediator Production

The RAW 264.7 cells were cultured in RPMI-1640 medium containing 10% BSA at a density of 1 × 10^5^ cells/ml. After being incubated overnight, the cells were stimulated with 200 ng/ml of LPS (*E. coli* 055: B5) with or without 32 μM of peptide. 24 h after peptide addition, the levels of NO, TNF-α, and IL-6 in the culture supernatant were measured by a Griess reagent or Quantikine murine kits (R&D Systems, Minneapolis, MN, United States) based on the instruction of manufacturer, respectively.

### Protection of Mice From LPS-Induced Lethal Infection

The 6–8 week-old male BALB/c mice (purchased from Charles River Laboratories, Beijing, China) were used to investigate the protective effect *in vivo* of peptides on LPS-induced infection. All animals were raised under specific pathogen-free conditions and the protocol was authorized by the Animal Care Committee of Northeast Agricultural University. Mice were divided into four groups (12 mice per group). 1 h after a challenge with a lethal dose of LPS intraperitoneal injection (20 mg/kg⋅body weight), 4 groups of mice were injected intraperitoneally with 0.5 ml PBS, PMB (2.5 mg/kg⋅body weight), dCATH(1–16) or dCATH(5–20) (10 mg/kg⋅body weight). General behavior, onset and course of clinical disease and mortality of mice were observed every 12 h for a period of 7 days.

### Statistical Analysis

Data analysis was conducted by one-way ANOVA using SPSS 17.0 software. The statistical differences among the groups were compared using Student’s *t*-test.

## Results

### Peptide Characterization

The main physico-chemical parameters of these peptides were as shown in Table 1. ESI-MS results indicated that the true molecular weights of these peptides were very consistent with their theoretical values (Table 1), suggesting that these peptides were successfully synthesized. Except dCATH(5–16) with net charge of +3, all other peptides possessed the net positive charge ≥6. Online tool I-TASSER was used to predict the three-dimension structures of the peptides. All of the peptides showed obviously α-helix structures except that the shortest peptide dCATH(5–16) exhibited unordered conformation (Supplementary Figure S1).

**TABLE 1:** Amino acid sequences, charge, length, molecular weights, and the hydropathicity values of the peptides used in this study.

**Peptides**	**Sequence**	**Net charge**	**Theoretical MW (Da)**	**Measured MW(Da)^a^**	**Theoretical pI**	**GRAVY^b^**
dCATH	KRFWQLVPLAIKIYRAWKRR-NH_2_	+8	2628.26	2628.28	12.02	–0.535
dCATH-AA	KRFAQLVPLAIKIYRAAKRR-NH_2_	8	2397.95	2397.86	12.02	–0.265
dCATH(1–16)	KRFWQLVPLAIKIYRA-NH_2_	+5	2001.49	2001.58	11.01	0.194
dCATH(1–17)	KRFWQLVPLAIKIYRAW-NH_2_	+5	2187.71	2187.73	11.10	0.129
dCATH(1–18)	KRFWQLVPLAIKIYRAWK-NH_2_	+6	2315.88	2315.90	11.17	–0.094
dCATH(5–20)	QLVPLAIKIYRAWKRR-NH_2_	+6	2010.51	2010.53	11.73	–0.263
dCATH(4–20)	WQLVPLAIKIYRAWKRR-NH_2_	+6	2196.72	2196.74	11.73	–0.300
dCATH(3–20)	FWQLVPLAIKIYRAWKRR-NH_2_	+6	2343.89	2343.92	11.73	–0.128
dCATH(5–16)	QLVPLAIKIYRA-NH_2_	+3	1383.74	1383.77	9.99	0.800
dCATH(1–16)-4A	KRFAQLVPLAIKIYRA-NH_2_	+5	1886.36	1886.38	11.10	0.362
dCATH(5–20)-17A	QLVPLAIKIYRAAKRR-NH_2_	+6	1895.37	1895.39	11.73	–0.094

*^*a*^Measured MW: The molecular weight (MW) was measured by ESI-mass spectroscopy (MS). ^*b*^GRAVY: The grand average hydropathy value of the peptides.*

### Hemolytic Activity

**FIGURE 1:**
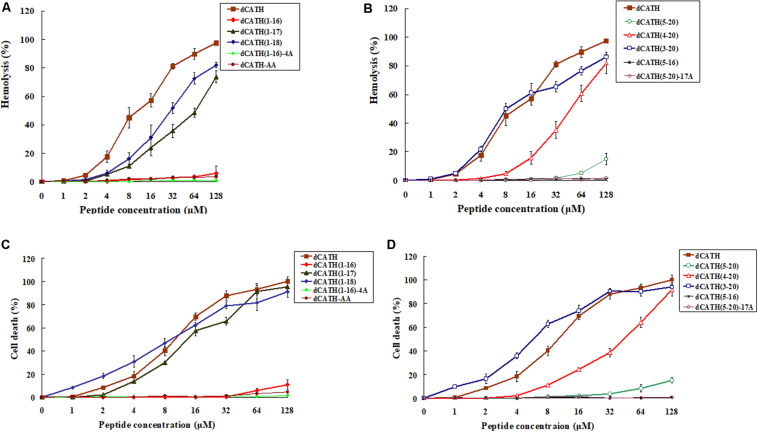
Hemolytic and toxicity activity of the peptides. **(A,B)** Hemolytic activity against hRBCs. **(C,D)** Toxicity activity against RAW264.7 cells. Hemolytic activity was evaluated by incubating individual peptides in serial 2-fold dilutions with freshly isolated hRBCs at 37°C for 1 h, followed by measuring the released hemoglobin at 405 nm. For negative and positive controls, hRBCs in PBS and 0.1% Triton X-100 were used, respectively. Toxicity activity of the peptides against RAW264.7 was determined using MTT method. The peptides were incubated with RAW264.7 in a 96-well microtiter plate. After incubation with MTT, the supernatant was discarded and DMSO was added and the optical density (OD) at 570 nm was measured.

The hemolytic activity of these peptides against hRBCs was measured, and the results were as shown in Figures 1A,B. The parent peptide and four derived analogs, dCATH(1–17), dCATH(1–18), dCATH(3–20), dCATH(4–20) showed obvious hemolytic activity. The hemolytic activities of dCATH(1–16), dCATH(1–17), dCATH(1–18), dCATH(5–20), and dCATH(4–20) were all less than that of the parent peptide dCATH. Especially, dCATH(1–16) and dCATH(5–20), which only contained one Trp residue (W4 or W17), showed very significantly reduced hemolytic activities compared with the parent peptide and derivatives containing two Trp residues. Even at the highest concentration of 128 μM, the hemolytic rates of the two peptides were only 6.02 and 14.80%, respectively. And then, four peptides that both Trp residues were replaced or diminished, dCATH(5–16), dCATH(1–16)-4A, dCATH(5–20)-17A, and dCATH-AA, showed negligible hemolytic activity.

### Cytotoxicity

The peptide cytotoxicity to RAW 264.7 cells was further examined by the MTT viability assay. As shown in Figures 1C,D, the parent peptide dCATH and four derived peptides, which contained two Trp residues (W4, W17), all showed high cytotoxicity. dCATH(1–17), dCATH(1–18), dCATH(4–20), dCATH(3–20) and dCATH almost eliminated all living cells, with the cell survival rates less than 10% at 128 μM. dCATH(1–16) only kept very weak cytotoxicity with the cell death of 5.80 or 10.91% at the concentration of 64 or 128 μM. dCATH(5–20) also displayed significantly reduced cytotoxicity compared with the parent peptide dCATH. The cell death rates of dCATH(5–20) were 8.30 and 15.10% at the concentration of 64 and 128 μM, respectively. In addition, other four peptides not containing any of two Trp residues (W4, W17) did not show cytotoxicity against RAW 264.7 cells.

### Antimicrobial Activity

The peptide MIC values against the tested bacteria are summarized in Table 2. Six dCATH truncated peptides containing Trp residue showed potent antibacterial activity against all tested bacteria similar with the parent peptide dCATH, with MIC values ranging from 1 to 8 μM. However, four dCATH derived peptides not containing Trp residue showed significantly reduced antimicrobial activity compared with dCATH. The GM reflected the therapeutic effect of the peptides against tested bacterial strains. As shown in Table 2, five dCATH truncated peptides, dCATH(1–17), dCATH(1–18), dCATH(5–20), dCATH(4–20), and dCATH(3–20), showed lower GM values compared with the parent peptide, which demonstrated that these peptides possessed higher antimicrobial activity across the bacterial species than dCATH. dCATH(1–16) had the highest TI value of 52.46, an improvement of 47.49 compared to dCATH. dCATH(5–20) also had high TI value of 39.02. Except for dCATH(1–16) and dCATH(5–20), all dCATH derivatives had poor cell selectivity although they showed slightly higher TI than dCATH. These results showed that dCATH(1–16) and dCATH(5–20) had higher cell selectivity toward bacterial cells than hRBCs, implying a wider therapeutic window.

**TABLE 2:** MIC, MHC, and TI of the peptide dCATH and its derivatives.

**Peptides**	**MIC (μM)^a^**							**GM (μM)^b^**	**MHC (μM)^c^**	**TI^d^**
	***E. coli* ATCC 25922**	***E. coli* UB1005**	***S. pullorum* C79-13**	***S. typhimurium* ATCC 14028**	***S. aureus* ATCC 29213**	***S. epidermidis* ATCC 12228**	***E. faecalis* ATCC 29212**			
dCATH	2	2	4	8	4	4	4	3.62	4	1.10
dCATH-AA	32	16	32	64	64	64	32	39.01	256	6.56
dCATH(1–16)	2	4	4	4	8	8	8	4.88	256	52.46
dCATH(1–17)	1	1	4	4	2	4	2	2.21	8	3.62
dCATH(1–18)	1	2	2	2	2	2	2	1.81	8	4.42
dCATH(5–20)	1	4	8	4	2	4	4	3.28	128	39.02
dCATH(4–20)	1	4	4	2	2	4	2	2.44	16	6.58
dCATH(3–20)	1	2	2	4	2	1	2	1.81	4	2.21
dCATH(5–16)	64	128	¿128	¿128	¿128	¿128	¿128	190.21	256	1.35
dCATH(1–16)-4A	16	32	64	64	128	64	32	47.55	256	5.38
dCATH(5–20)-17A	64	¿128	¿128	64	32	32	32	70.66	256	3.62

*^*a*^Minimum inhibitory concentrations (MICs) were determined as the lowest concentration of the peptides that inhibited bacteria growth. ^*b*^When no detectable antimicrobial activity was observed at 128 μM, a value of 256 μM was used to calculate the peptide GM. ^*c*^MHC is the minimum hemolytic concentration that caused 10% hemolysis of human red blood cells (hRBC). 256 μM was used as MHC of a peptide when hemolysis rate was less than 10% at the peptide concentration of 128 μM. ^*d*^TI is the ratio of the MHC to the geometric mean of MIC (GM). Larger values indicate greater cell selectivity.*

### Salt Sensitivity

Salt tolerance is very crucial for AMPs to exhibit antimicrobial activity *in vivo*. The salt sensitivity of the two peptides, dCATH(1–16) and dCATH(5–20), was investigated in the presence of different physiological concentrations of salt ions. As shown in Table 3, salt ion concentrations had little effect on the activities of dCATH(1–16) and dCATH(5–20) against the *E. coli* ATCC25922 and *S. aureus* ATCC 29213. Even some ions showed promoting effect on their antimicrobial activities, such as Ca^2+^ and Zn^2+^. These results demonstrated that the two peptides maintained effectively antimicrobial activity in physiological salt concentrations.

**TABLE 3:** MIC values of peptides in the presence of physiological concentration salts.

**Strains and peptides**	**Control^a^**	**NaCl^a^**	**KCl^a^**	**NH_4_Cl^a^**	**MgCl_2_^a^**	**ZnCl_2_^a^**	**CaCl_2_^a^**	**FeCl_3_^a^**
***E. COLI* ATCC25922**								
dCATH(1–16)	2	2	2	2	4	2	1	2
dCATH(5–20)	1	1	1	1	2	1	1	1
***S. AUREUS* ATCC 29213**								
dCATH(1–16)	8	8	8	8	8	4	4	8
dCATH(5–20)	2	2	2	2	2	2	2	2

*^*a*^The final concentrations of NaCl, KCl, NH_4_Cl, MgCl_2_, CaCl_2_, ZnCl_2_, and FeCl_3_ were 150 mM, 4.5 mM, 6 μM, 1 mM, 2.5 mM, 8 μM, and 4 μM, respectively. The control MIC values were determined in the absence of these physiological salts.*

### Fluorescence Spectroscopy

When Trp containing peptide binds to liposomes of prokaryotic or eukaryotic biofilm, the maximum emission wavelength of peptide will move to a shorter wavelength, which is called blue shift. In this study, the liposomes of PC/PG (7:3, w/w) and PC/CH (8:1, w/w) were prepared to mimic bacterial and eukaryotic membrane, respectively. The binding ability between peptides with cytoplasmic membrane was evaluated according to the interaction between peptides with PC/PG or PC/CH liposomes. The Trp blue shifts of these peptides in membrane-stimulated vesicles were as shown in Table 4. dCATH and its four analogs, dCATH(1–17), dCATH(1–18), dCATH(4–20), dCATH(3–20), exhibited obvious blue shifts in the presence of negatively charged liposomes (PC/PG) (22–27 nm) and neutral liposomes (PC/CH) (16–22 nm), which suggested that Trp residues in these peptides were involved in the membrane hydrophobic portion. dCATH(1–16) and dCATH(5–20) showed significant blue shifts in the PC/PG liposomes (25 and 24 nm), whereas had very low blue shifts in the PC/CH liposomes (3 and 2 nm). The data demonstrated that the interaction of the two peptides with the prokaryotic cell membrane was stronger than that with the eukaryotic cell membrane, and were in accordance with the facts that dCATH(1–16) and dCATH(5–20) had improved cell selectivity.

**TABLE 4:** Trp emission maxima of the peptides in HEPES buffer (pH 7.4, 10 mM HEPES, 150 mM NaCl), or in the presence of PC/PG (7:3, w/w), and PC/CH (8:1, w/w) liposomes.

**Peptides**	**Tryptophan emission maxima (nm)**		
	**HEPES buffer**	**PC/PG**	**PC/CH**
dCATH	350	324 (26)^a^	329 (21)
dCATH(1–16)	346	321 (25)	344 (2)
dCATH(1–17)	348	326 (22)	332 (16)
dCATH(1–18)	348	325 (23)	331 (17)
dCATH(5–20)	347	323 (24)	343 (4)
dCATH(4–20)	348	326 (22)	332 (16)
dCATH(3–20)	348	321 (27)	322 (22)

*^*a*^Blue shift in emission maximum in parentheses.*

### Cytoplasmic Membrane Electrical Potential

**FIGURE 2:**
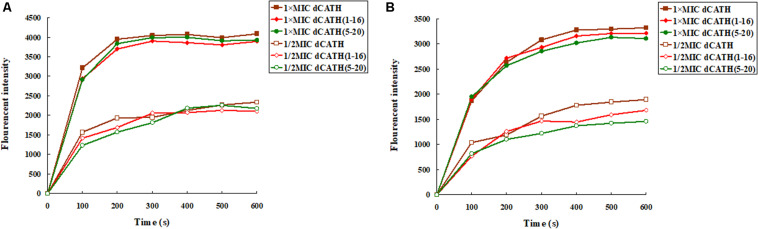
*Cytoplasmic membrane depolarization* ability of the peptides. *Cytoplasmic membrane depolarization* ability of the peptides was measured using the membrane potential-sensitive dye diSC3–5. *E. coli* ATCC 25922 and *S. aureus* ATCC 29213 were treated by 1 × MIC and 1/2 MIC peptides, and the fluorescent intensity was monitored at an excitation wavelength of 622 nm and an emission wavelength of 670 nm as a function of time. **(A)** The membrane potential variation of *E. coli* ATCC 25922 treated by peptides, **(B)** the membrane potential variation of *S. aureus* ATCC 29213 treated by peptides.

Once the cytoplasmic membrane is permeabilized and disrupted, the membrane potential will dissipate. Then, the diSC_3_-5 can be released into the buffer, resulting in enhanced fluorescence. As shown in Figure 2, the cytoplasmic membrane depolarization of the two bacterial cells treated with the peptides appeared in dose- and time-dependent manner. dCATH, dCATH(1–16), and dCATH(5–20) leaded to rapid increase in the fluorescence intensity. However, dCATH(1–16) and dCATH(5–20) induced lower increase in fluorescence intensity compared with dCATH.

### Membrane Permeability

**FIGURE 3:**
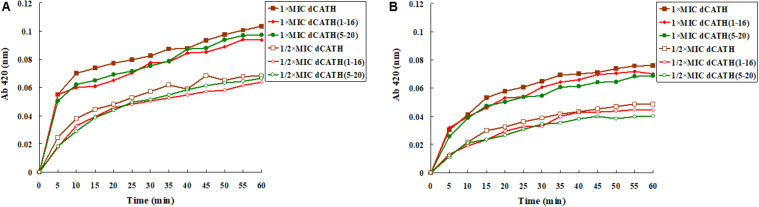
Cytoplasmic membrane variation of *E. coli* ATCC 25922 and *S. aureus* ATCC 29213 treated by peptides. ONPG was cleaved by cytoplasmic β-galactosidase of *E. coli* ATCC 25922 and *S. aureus* ATCC 29213 treated by 1 × MIC of dCATH, 1 × MIC of dCATH (1–16), 1 × MIC of dCATH (5–20), 1/2 × MIC of dCATH, 1/2 × MIC of dCATH (1–16), 1/2 × MIC of dCATH (5–20), respectively. Hydrolyzate (ONP) was measured spectroscopically at absorbance of 420 nm as a function of time. **(A)** *E. coli* ATCC 25922, **(B)** *S. aureus* ATCC 29213.

ONPG will enter the cytoplasm if permeabilization of the cell membrane is induced by peptide. ONPG can be degraded by β-galactosidase to produce yellow *o*-nitrophenol with an absorbance of 420 nm. Figure 3 showed that dCATH, dCATH(1–16) and dCATH(5–20) induced a rapid increase in inner membrane permeability of bacterial cells at their 1 × MIC concentrations within 60 min, and the absorption values for these peptides at 1/2 × MIC were lower significantly than those at 1 × MIC. The inner membrane permeability of dCATH(1–16) and dCATH(5–20) was reduced compared with that of dCATH.

### SEM and SEM

**FIGURE 4:**
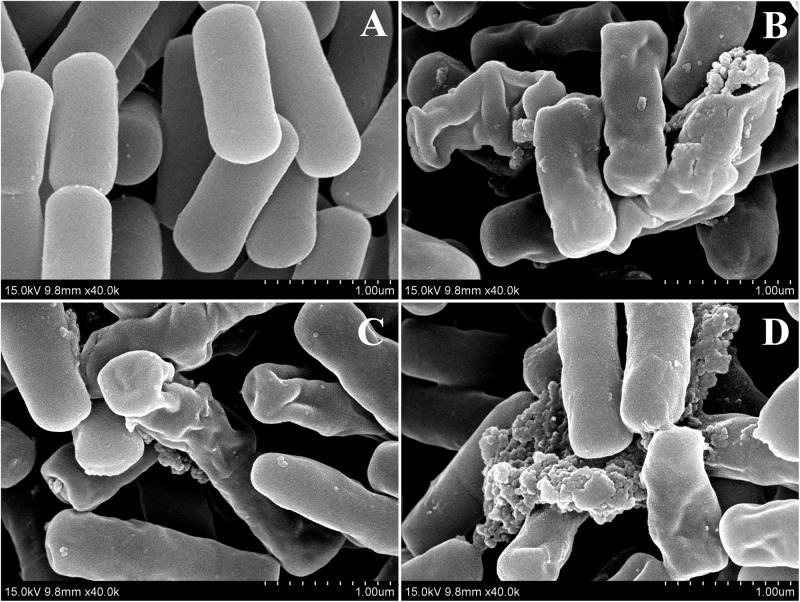
SEM micrographs of *E. coli* ATCC 25922 treated by peptides at 1 × MICs. **(A)** Control, **(B)** dCATH for 60 min, **(C)** dCATH(1–16) for 60 min, and **(D)** dCATH(5–20) for 60 min. Control was processed without peptides.

Two peptides, dCATH(1–16) and dCATH(5–20), were selected to further investigated their antibacterial mechanism in the following SEM and TEM experiments. As shown in Figure 4, great biomembrane damage of *E. coli* ATCC 25922 was caused by the two peptides at their 1 × MICs for 60 min. The bacterial cells in the control had a plump shape and smooth surface, while the morphology of bacterial cells exposed to the peptides showed obviously changed, as evidenced by the appearances of membrane shrinkage, distortion and breakage.

**FIGURE 5:**
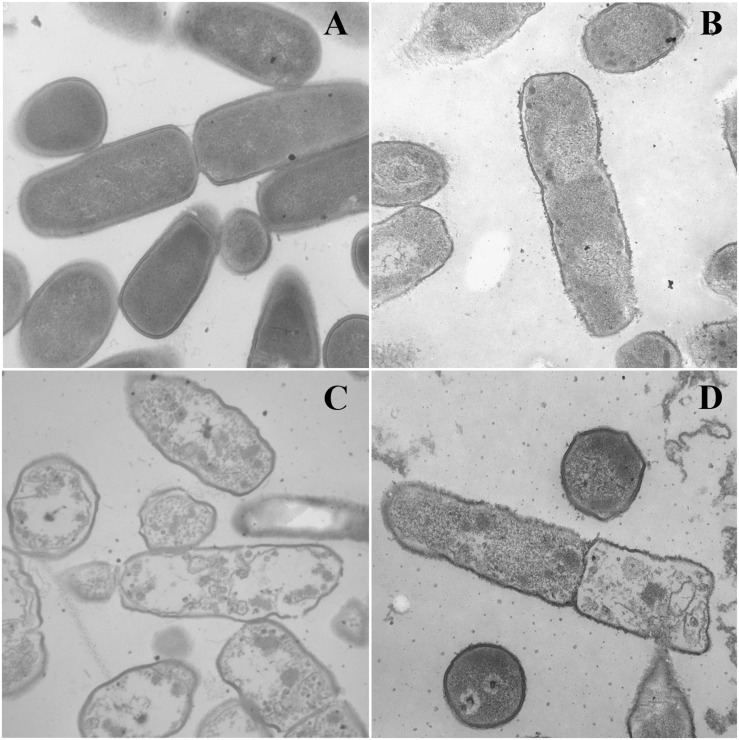
TEM micrographs of *E. coli* ATCC 25922 treated by peptides at 1 × MICs. **(A)** Control, **(B)** dCATH for 60 min, **(C)** dCATH(1–16) for 60 min, and **(D)** dCATH(5–20) for 60 min. Control was processed without peptides.

Transmission electron microscopy was used to observe alteration of the morphology and intracellular of bacterial cells treated with peptides. As shown in Figure 5, smooth cell surface and dense internal structure were observed for *E. coli* ATCC 25922 cells without peptide treatment. Cytoplasmic component was distributed well and filled the entire space surrounded by the bacterial wall. However, *E. coli* ATCC 25922 cells treated with peptides showed significant membrane rupture and release of cytoplasmic contents (Figure 5). Compared with the control, the cytoplasmic contents in cells treated with peptides showed more aggregation and coagulation.

### LPS Binding Ability

**FIGURE 6:**
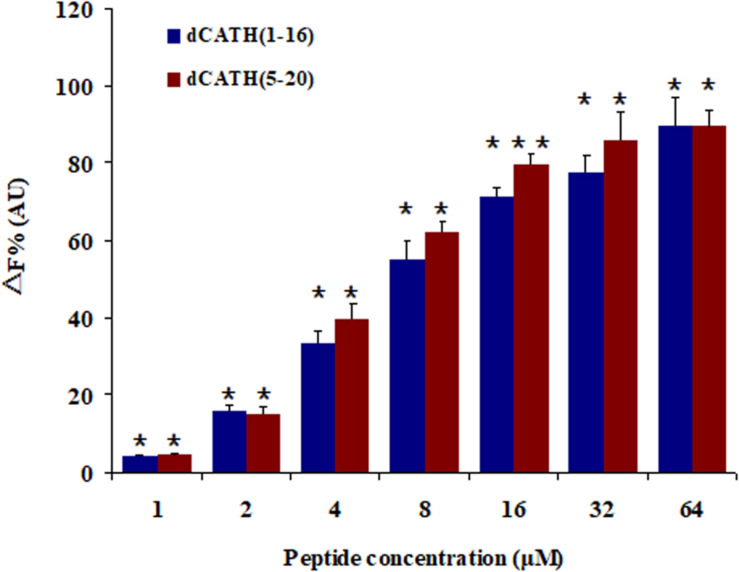
Peptide binding affinity to LPS from *E. coli* 055:B5. The combining ability of dCATH (1–16) and dCATH (5–20) (1, 2, 4, 8, 16, 32, and 64 μM) with LPS. The graphs were derived from average values of three independent trials. Means in the same concentration with different superscript are very significant difference (*P* < 0.01).

A fluorescence based displacement determination using BC was carried out to measure the binding ability of peptide to LPS, with PMB as a positive control. As shown in Figure 6, the two peptides produced obvious LPS-binding ability with dose-dependent manner. When the concentration was greater than 16 μM, the LPS-binding efficiencies of the two peptides were higher than 70% of PMB binding activity.

### Inhibition of Pro-inflammatory Mediator in RAW 264.7 Cells by LPS-Stimulation

**FIGURE 7:**
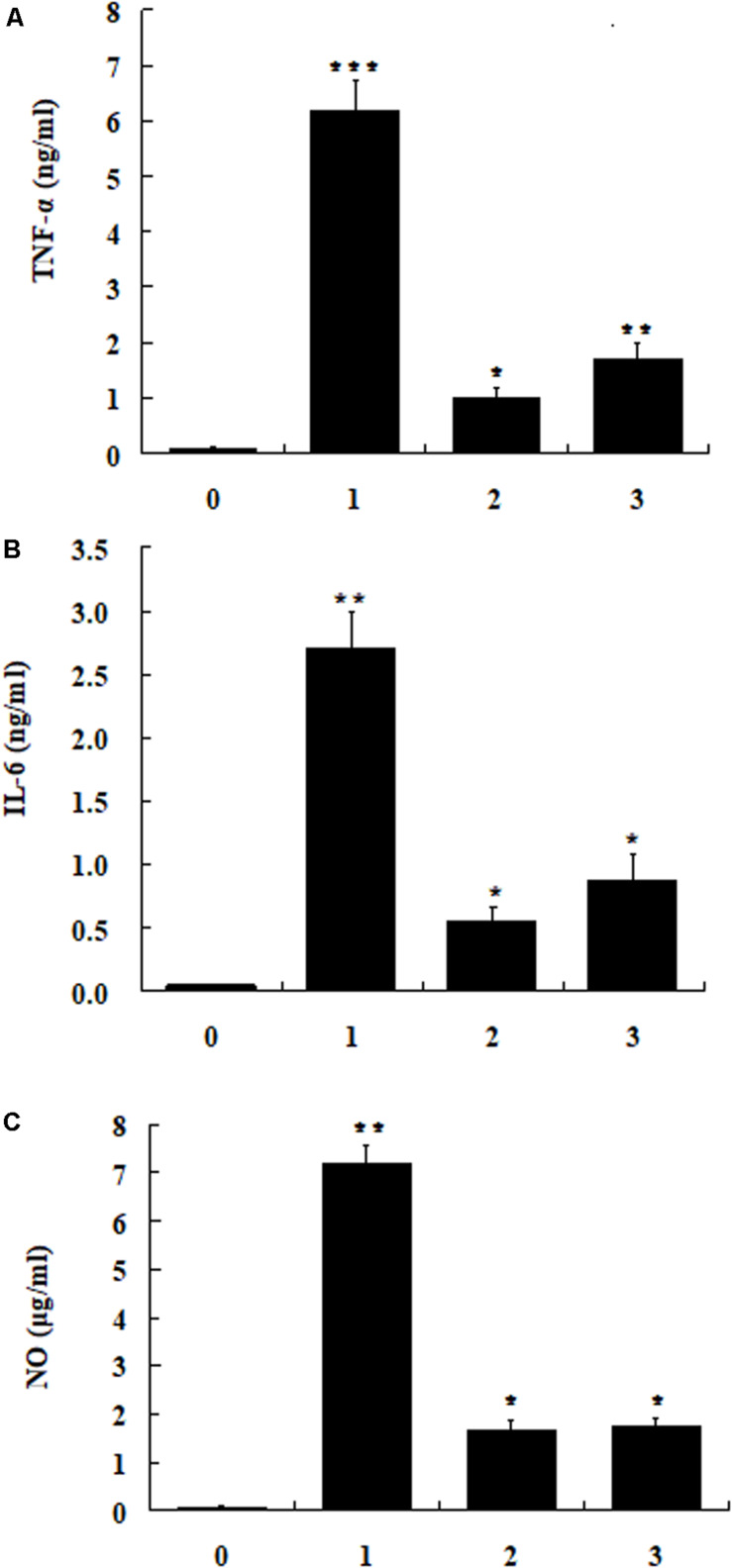
The RAW264.7cells were treated with LPS (200 ng/ml) and the peptide dCATH(1–16) or dCATH(5–20) at the concentrations of 32 μM for 24 h. TNF-α **(A)** and IL-6 **(B)** production was measured with an ELISA method. NO **(C)** production was assayed using Griess reagent. (0), (1), (2), and (3) were control, LPS treated, LPS + dCATH(1–16) treated and LPS + dCATH(5–20) treated, respectively. All bar graphs represent the mean ± SEM of three independent experiments. Means in the same concentration with different superscript are very significant difference (*P* < 0.01).

The cytotoxic activities of dCATH(1–16) and dCATH(5–20) were detectable at 64 μM or greater concentrations, and were negligible below 32 μM (Figure 2). In addition, 32 μM of dCATH(1–16) and dCATH(5–20) showed strong LPS-binding ability (Figure 6). Therefore, the subsequent experiment was conducted at a non-toxic concentration of 32 μM. The inhibition of the two peptides on the production of representative pro-inflammatory mediators in the RAW264.7 cells stimulated by LPS was evaluated. As shown in Figure 7, the peptides significantly inhibited the levels of TNF-α, IL-6 and NO at the concentration of 32 μM. dCATH(1–16) suppressed TNF-α by 88.7%, IL-6 by 79.6% and NO by 76.8%. dCATH(5–20) suppressed TNF-α by 72.3%, IL-6 by 67.8% and NO by 75.4%. These results indicated that the two peptides exerted anti-inflammatory effect through inhibiting pro-inflammatory factor release of LPS-induced macrophages.

### Protection of Mice From LPS-Induced Lethal Infection

**FIGURE 8:**
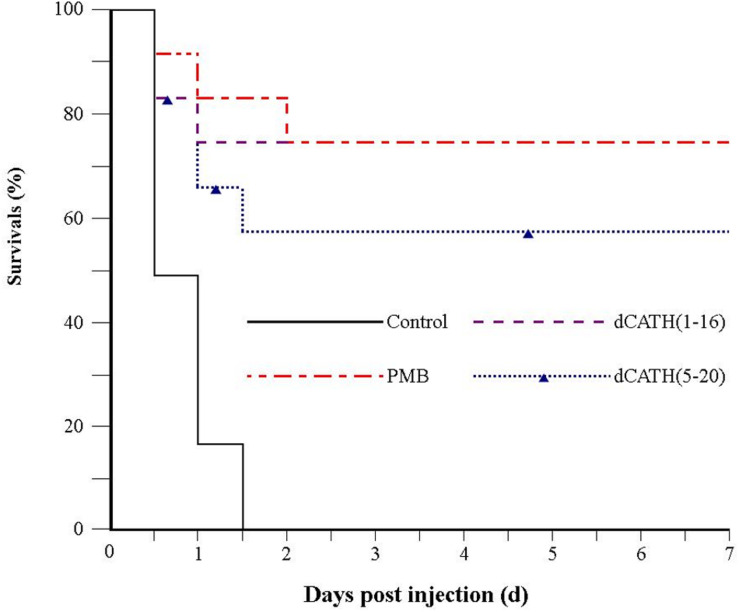
Survival of mice from LPS-induced lethal infection by dCATH(1–16) or dCATH(5–20). Mice were subjected to a lethal dose of LPS, followed by i.p. injection of CATH(1–16) or dCATH(5–20) at 10 mg/kg or an equal volume of PBS as controls (*n* = 12). Survival was recorded every 12 h over 7 days.

Because of desirable antimicrobial and anti-inflammatory activities, dCATH(1–16) and dCATH(5–20) were further evaluated for their protective effects on LPS induced lethal infection in mice. After being injected with a lethal dose of LPS (20 mg/kg⋅body weight), four group mice were then injected with PBS alone, PMB, dCATH(1–16) and dCATH(5–20) in PBS, respectively. Mice were observed for the health and survival rate for 7 days. As shown in Figure 8, all twelve mice in control group died within 36 h after LPS injection. In addition, injection with PMB or the two peptides delayed the onset of disease of mice with less severe symptoms of drowsiness, furry folds, and anorexia. 75% (9/12 mice) in dCATH(1–16) group and 58.3% (7/12 mice) in dCATH(5–20) group survived and showed normal behavior by day 7, displaying potential application of the two peptides for LPS infection treatment.

## Discussion

Antimicrobial peptides have been widely recognized as potential novel drugs because of their broad-spectrum antimicrobial activity including enveloped viruses and fungi (Ramanathan et al., 2002), and reduced bacterial resistance (Teixeira et al., 2012). The AMPs of CATH family have roles in wound repair, cell chemotaxis, inhibition of tissue damage caused by LPS, promotion of angiogenesis and other important activities (Wuerth and Hancock, 2011). Many natural AMPs have a high cytotoxicity and hemolytic activity. To improve the cell selectivity of AMPs, truncation or residue-substitution of natural AMPs is often been considered as an effective method to design and develop novel analogs of AMPs (Jacob et al., 2013; Jittikoon et al., 2015; Qu et al., 2016). In this study, several truncated and Trp-replaced analogs of dCATH were designed, and their activity *in vitro* and action mechanism were also investigated.

Binding of AMPs to the bacterial cell membrane mainly depends on electrostatic attraction between AMPs with net cationic charges and negatively charged components of bacterial membrane. A sufficiency of net cationic charge is one of the most crucial factors for antimicrobial activity of AMPs. However, AMPs with increased positive charge do not show an increase in antimicrobial activity above a certain threshold (Dathe et al., 2001). As shown in Table 2, the derivatives of dCATH displayed different antimicrobial activity to the tested bacteria. The peptide dCATH with + 8 net charges did not exhibit higher antimicrobial activity than its analogs with lower net charges, such as dCATH(1–17), dCATH(1–18), dCATH(3–20), dCATH(4–20), and dCATH(5–20). On the other hand, the antimicrobial activities of dCATH(1–16) and dCATH(1–17) with + 5 net charges were not significantly reduced compared with that of dCATH. These results demonstrated that five positive charges in the peptide sequence of dCATH were enough to cause antimicrobial activity.

As amino acid residues with positive charge, Arg (R) and Lys (K) residues endow AMPs with hydrogen bonding properties and cationic charges essential to binding to the negatively charged surface of microbial lipid membranes. AMPs with more cations can be produced by substituting with Arg or Lys residue. With the increase of positive charge, interaction with bacterial membrane and antimicrobial activity of AMPs were enhanced (Chan et al., 2006; Junkes et al., 2011; Han et al., 2016). Arg and Lys are rich in the N- and C-terminuses of the peptide dCATH. However, all the dCATH analogs removing Arg or Lys residues in this study exhibited similar antimicrobial activity with the parent peptide. These results indicated that deletion of the structures of KR- at the N-terminus or -KRR at the C-terminus of dCATH did not attenuate its antimicrobial activity, and more Arg and Lys residues in the peptide have no effect on its antimicrobial activity improvement.

Trp in many natural AMPs is often in relatively high proportion and always has significant roles in antimicrobial activity (Chan et al., 2006). As a hydrophobic amino acid, Trp can anchor AMPs to the bacterial membrane mainly depending on aromatic side chain of Trp, which can affect the interface region of lipid bilayer and disturb the internal structure of cell membrane (Chan et al., 2006; Bi et al., 2013). Generally, more Trp content in AMPs contributes to stronger antimicrobial activity. However, the number of Trp residue has a pivotal role in the cell selectivity of AMPs. Strom et al. (2002) showed that three or more Trp residues ensured AMPs with a good antimicrobial activity, while four or more Trp residues increased cytoxicity against hRBCs. Much work has been done to achieve ideal bioactive AMPs by the means of *de novo* design of simple model AMPs or modification/optimization of natural AMPs using Trp residue (Deslouches et al., 2005; Pasupuleti et al., 2009; Junkes et al., 2011; Rydberg et al., 2012; Torcato et al., 2013). As a hydrophobic amino acid, alanine (Ala) has the same structure of carbon chain with the hydrophobic Trp except aromatic side chain. There are two Trp residues in the structure of dCATH (W4 and W17). Trp-deleted or Ala-replacing derived peptides in this study were employed to investigate the Trp role in the dCATH. Hemolytic activity and cytotoxicity of these peptides were assessed by measuring the survival and damage of normal cells treated with these peptides. As shown in Table 2, the two peptides, dCATH(1–16) and dCATH(5–20), which only have one of Trp residues (W4 and W17), showed similar antimicrobial activity with the parent peptide and other derivatives containing two Trp residues, while possessed much lower hemolytic and cytotoxic activity than them (Figure 1). Moreover, hemolytic and cytotoxic activity was almost disappeared and antimicrobial activity was also significantly reduced when the two Trp residues in dCATH were deleted. These results demonstrated that the presence of Trp was a critical factor for the antimicrobial efficacy, but too much number of Trp residue was a major cause with high hemolysis rate and cytotoxicity, which was consistent with previous study of Strom et al. (2002). One Trp residue is essential and enough to maintain the antimicrobial activity of dCATH in this study. However, appropriate number of Trp residue needs to be further investigated for other AMPs containing Trp residue.

The therapeutic potential of AMPs is highly dependent on the cell selectivity of these peptides, which can effectively kill bacterial cells without obvious cytotoxicity to mammalian cells. Cell selectivity also evaluates the ability of AMPs to distinguish any pathogen from host cells. This concept is defined by the TI, and a high TI thus represents two priority properties of AMPs as below: a high MHC (low hemolysis) and a low MIC (high antimicrobial activity). In another word, larger TI value indicates higher cell selectivity. Table 2 summarized the TI values of these peptides in this study. dCATH(1–16) and dCATH(5–20) showed much higher TI than others. The Trp fluorescence assay also demonstrated that the binding preference of the two peptides to liposomes of simulating the prokaryotic and eukaryotic cell membranes was consistent to their properties of cell selectivity. Deletion one of the two Trp residues (W4 and W17) in dCATH did not significantly influence to be combined with anionic liposomes, but significantly affected to be combined neutral liposomes, which is consistent with the cell selectivity characterization of dCATH(1–16) and dCATH(5–20). These data also demonstrated that two Trp residues of dCATH were very important to its hemolytic activity and cytotoxicity, and deletion one of them can significantly improve its cell selectivity. The two peptides caused 6.02 and 14.80% hemolysis of hRBCs at the highest concentration of 128 μM, respectively. The TI values of the two peptides, which were more important and comprehensive to evaluate AMPs function as antimicrobial agents, were 52.46 and 39.02, respectively. The high TI values suggested that the two peptides, dCATH(1–16) and dCATH(5–20), could be developed as novel antimicrobial agents.

The first step for AMPs to play antimicrobial activity mainly depends on electrostatic interaction and binding to the anionic components on the bacteria cell wall. The carboxyl and phosphate groups of the teichoic acids and carboxyl groups of peptidoglycan in that of Gram-positive bacteria (Omardien et al., 2016) or lipopolysaccharide in that of Gram-negative bacteria (Ebenhan et al., 2014) endows the overall bacteria with negative charges, and has strong electrostatic attraction to AMPs with positive charges. AMPs bind to the bacterial cell wall by electrostatic attraction, and then contact with the cell membrane of bacteria by electrostatic action and interact with the lipid bilayer (Nguyen et al., 2011). One of the means that AMPs kill bacteria is to destroy cell membrane (Brogden, 2005; Sato and Feix, 2006), and this also explains why AMPs are less possibility to cause drug resistance of bacteria. The cytoplasmic membrane of bacteria is largely composed of hydroxyl phospholipids with negative charge at physiological pH value (Teixeira et al., 2012), which is beneficial for the cationic AMPs binding. The peptide molecule is located and inserted into the lipid bilayer of the cell membrane, resulting in damage to membrane permeability and integrity or formation of pore/ion channel, simultaneously accompanied by the collapse of membrane potential (Sato and Feix, 2006; Fjell et al., 2012). In this study, measurement of cell membrane permeability and potential was carried out. As expected, the selected peptides, dCATH(1–16), dCATH(5–20) and the parent peptide dCATH, increased the permeability of the inner membrane to ONPG at concentration of 1 × MIC (Figure 3). The potential across the bacterial cytoplasmic membrane was dissipated by the membrane destruction (Figure 2) that would lead to leakage of cell cytoplasm and cell death.

To further investigate the interaction between the peptides and membrane, *E. coli* cells treated with dCATH(1–16), dCATH(5–20), and dCATH were observed by SEM and TEM. As shown in Figure 4, the membrane blebbing and withering was caused after treatment with these three peptides because the fluid leaked into the cytoplasm, which indicated that these peptides may lead to destruction of cell membrane, change of membrane permeability and leakage of cytoplasmic content. TEM analysis also provided morphological evidence that these peptides possessed strong membrane permeability (Figure 5). Given the above, these results demonstrated that the three peptides would selectively combine with cell membrane components, alter the membrane permeability, damage the membrane of bacterial cells, and finally kill bacteria. Overall, this is the first study on the structure-activity relationship and novel derived peptides of the peptide dCATH. These results obtained in this study could be useful in designing novel AMPs with short amino-acid sequence as therapeutic agents and correlating its function as antimicrobial activity and cell membrane permeabilization.

Besides AMPs cell selectivity, another important issue in drug development of AMPs is their stability. Generally, the presence of salt ions may cause various negative influences such as degeneration or reduced activity of AMPs. Interestingly, the antimicrobial activities of dCATH(1–16) and dCATH(5–20) were not attenuated, or even increased in the presence of Ca^2+^ and Zn^2+^ (Table 3). At high concentrations, divalent cations gradually increase the rigidity of cell membrane by electrostatic interactions with negatively charged phospholipids, thus slowly impeding the formation of pores. However, the existence of a small amount of divalent cations can promote the binding of AMPs to the bacterial membrane (Aquila et al., 2013), which may be the reason why Ca^2+^ and Zn^2+^ ions enhanced the activity of the two peptides in this study. Inflammation is a defense response of host tissue to injury, as well as a pathological process including damage and resistance to damage. Severe inflammatory responses can cause sepsis. Extensive interest has been focused in the suppressing inflammatory responses by AMPs. It is well known that NO, TNF-α, and IL-6 are the major inflammation mediators. Overproduction of NO, TNF-α, and IL-6 causes inflammatory reactions harmful to organisms. Many AMPs exert play anti-inflammatory roles through binding to LPS, such as LL-37 (Scott et al., 2011), indolicidin (Nan et al., 2009) and Fowl-1 (8–26)-WRK (Rajasekaran et al., 2019). dCATH(1–16) and dCATH(5–20) possess α-helical structure with net positive charge, which is favorable to the combination with LPS with negatively charge. It is not surprising that dCATH(1–16) and dCATH(5–20) showed anti-LPS capabilities (Figure 6) and anti-inflammatory properties. In the present study, dCATH(1–16) and dCATH(5–20) significantly inhibited RAW 264.7 cells stimulated by LPS to produce three inflammatory mediators (Figure 7). Particularly important, injection of the two peptides led to a drastic advantage in survival compared with the untreated mice (Figure 8). These results showed that dCATH(1–16) and dCATH(5–20) showed significant anti-inflammatory activity, which can effectively suppress inflammatory reaction and sepsis.

In this study, the results presented herein demonstrated that the number of Trp residue had a pivotal role in the antimicrobial efficiency, hemolytic and cytotoxic activity of the peptide dCATH. One Trp presence was essential and competent for dCATH to maintain the antimicrobial activity. However, co-exist of two Trp residues (W4 and W17) was the major cause of dCATH with high hemolytic activity and cytotoxicity. Deletion of one Trp residue significantly increased the cell selectivity of the peptide. Two screened peptides, dCATH(1–16) and dCATH(5–20), and the parent peptide dCATH can penetrate bacterial cell membrane, depolarize cell membrane, destroy the integrity of cell membrane, lead to leakage of cytoplasm and cell death. dCATH(1–16) and dCATH(5–20) significantly inhibited the production of IL-6,TNF-α and NO in RAW 264.7 cells stimulated by LPS, and protected mice against endotoxic mortality with respect to LPS challenge. Therefore, the two peptides, dCATH(1–16) and dCATH(5–20), had high cell selectivity and anti-inflammatory effect *in vitro* and *in vivo*, and present promising potential as antimicrobial agents for clinical application.

## Data Availability Statement

All datasets generated for this study are included in the article/Supplementary Material.

## Ethics Statement

The animal study was reviewed and approved by the Animal Care Committee of Northeast Agricultural University.

## Author Contributions

XF led experimental work and wrote the first draft of the manuscript. SJ performed the antimicrobial assay and salt sensitivity assay. MW performed the hemolytic assay and the cytotoxicity assay. QP and SJ performed the membrane permeability assay. CL reviewed and edited the manuscript. RL and YW performed SEM analysis. RL and HY performed TEM analysis. FL and YL performed experiments of RAW 264.7 cells and mice challenged with LPS.

## Conflict of Interest

The authors declare that the research was conducted in the absence of any commercial or financial relationships that could be construed as a potential conflict of interest.

## Footnotes

1 http://www.expasy.org/tools/protparam.html

2 http://aps.unmc.edu/AP/main.php

3 http://zhanglab.ccmb.med.umich.edu/I-TASSER/

## Supplementary Material

FIGURE S1 Three dimensional structure projections of dCATH and its derivatives.
