# Supplementary figures and images for: The Critical Role of Tryptophan in the Antimicrobial Activity and Cell Toxicity of the Duck Antimicrobial Peptide DCATH
Source: Front Microbiol. 2020 May 28;11:1146. doi: 10.3389/fmicb.2020.01146 (PMC7326067; doi:10.3389/fmicb.2020.01146)

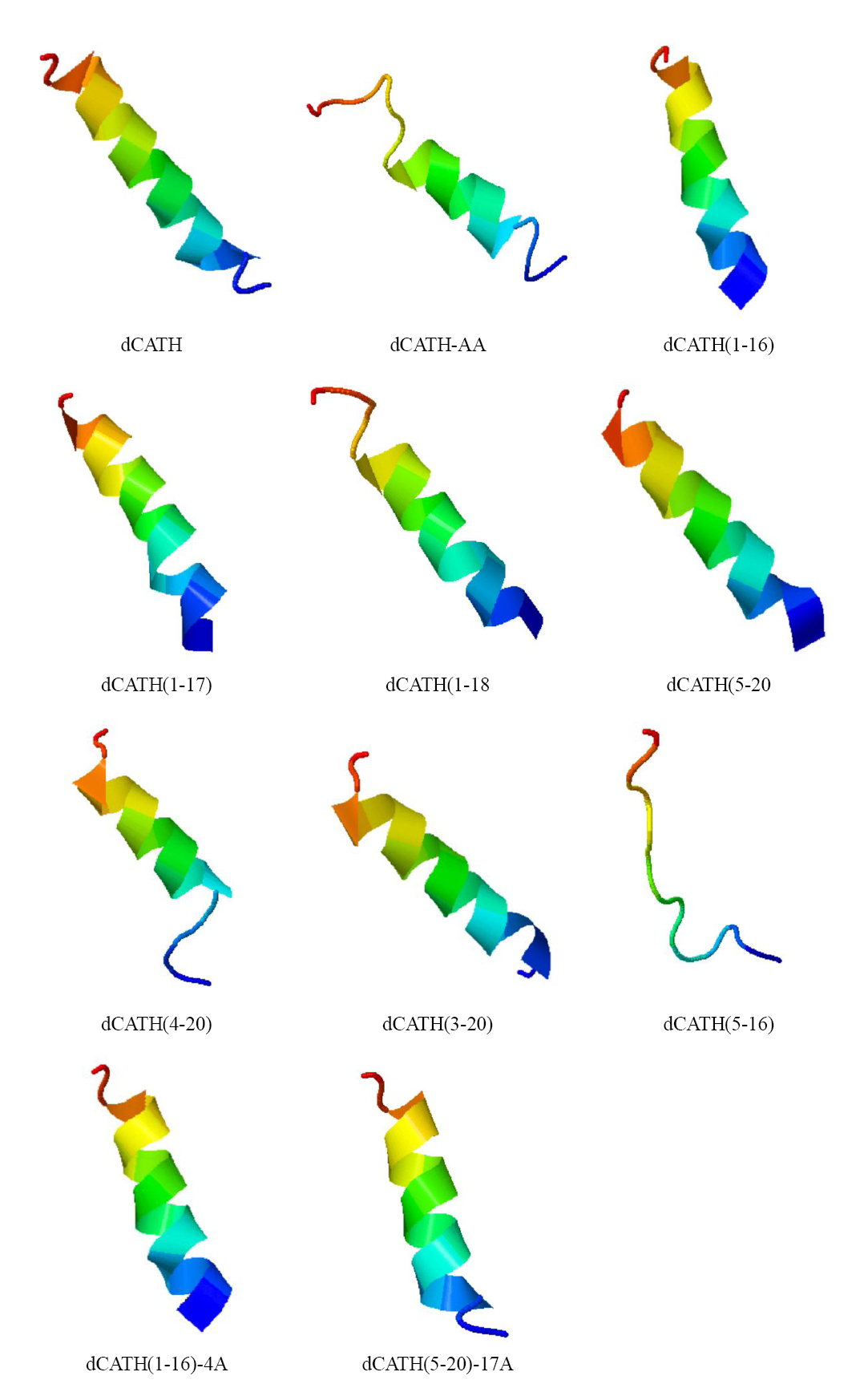

Supplement: FIGURE S1 — Three dimensional structure projections of dCATH and its derivatives. [file Image_1.TIF]
